# Detecting medial patellar luxation with ensemble deep convolutional neural network based on a single rear view image of the hindlimb

**DOI:** 10.1038/s41598-023-43872-7

**Published:** 2023-10-10

**Authors:** Juho Jung, Geonwoo Park, Gwanghyeon Kim

**Affiliations:** 1 Department of Applied Artificial Intelligence, Sungkyunkwan University, Seoul, Korea; 2 Elevenliter Inc., Samuiwon Startup Center, 26 Kyunghee-Daero, Dongdaemun-Gu, Seoul, Korea; 3 School of Computing, KAIST, Daejeon, Korea

**Keywords:** Zoology, Health care, Signs and symptoms, Engineering

## Abstract

Medial patellar luxation (MPL) is a common orthopedic disease in dogs, which predisposes elderly and small-breed dogs. Unlike in humans, diagnosis in the early course of the disease is challenging because symptoms and joint-pain expression in canines are vague. Herein, we introduced a deep-learning system to diagnose MPL using a single rear-view hindlimb image. We believe that this is the first attempt to build a deep-learning system to diagnose MPL based on image analysis. Notably, 7689 images were collected from 2653 dogs in 30 private animal clinics between July 2021 and July 2022. Model performance was compared with ResNet50, VGG16, VGG19, Inception-V3, and veterinarian performance. For performance comparison, a professional veterinarian with > 10 years of experience selected images of 25 normal dogs and 25 dogs with MPL. The proposed model showed the highest performance, with 92.5% accuracy, whereas human experts showed an average accuracy of 55.2%. Therefore, our model can diagnose MPL using only a single rear-view hindlimb image. Furthermore, to solve the image uncertainty caused by the input image noise, we used a one-class SVM and ensemble learning methods to ensure model robustness. Our study will help diagnose MPL in clinical settings using a single rear-view hindlimb image.

## Introduction

Patellar luxation (PL) is a common orthopedic condition in dogs that can cause lameness and secondary degenerative joint disease (DJD)^1–3^. PL occurs in medial and lateral directions (MPL and LPL, respectively) and is graded for severity on a 4-point scale. MPL is more common than LPL, with over 90% of small-breed dogs diagnosed with MPL^4^. The incidence of MPL was reported to be 12 times higher in small dogs than in large dogs^5^. The breeds most affected by PL include Maltese, Poodle, and Pomeranian^4,6^. Since PL is a progressive disease with a high potential to degrade the quality of life, it is of utmost importance to diagnose the disease early and take preventive measures^3^.

Physical examination and radiography are commonly used to diagnose PL, but these methods rely on the veterinarian’s subjective evaluation^7^. The quadriceps angle, used to diagnose and grade PL, is measured between two lines connecting three anatomic sites: the line connecting the anterior superior iliac spine and midpoint of the patella and the line connecting the tibial tuberate and midpoint of the patella^8^. This angle also represents the direction of the force of the quadriceps muscle^9^. The quadriceps angle is measured using diagnostic imaging methods, including radiography, computed tomography, and magnetic resonance imaging. Multiple studies have demonstrated a strong correlation between the quadriceps angle and PL, and the quadriceps angle is widely accepted as a reliable diagnostic index^8–10^.

With advances in technology, canine PL has been successfully diagnosed using a region with convolutional neural networks (R-CNN) deep learning model and 2832 radiographic images of the canine stifle joint^11^. However, this model requires radiographic images taken by veterinarians, which can only be obtained in veterinary clinics. In this study, we propose a deep-learning model that diagnoses PL using a single rear-view image of dogs without the need to visit veterinary clinics. To the best of our knowledge, this is the first deep-learning model to detect PL without diagnostic images. The performance of the model was compared with the diagnosis of veterinarians and gradient-weighted class activation mapping (Grad-CAM)^12^ was applied to visualize the key areas with representative features that are utilized for the classification process.

## Methods

### Data collection and labeling

In this study, we enrolled 30 private animal clinics to collect images of dogs. From July 2021 to July 2022, the clinics collected 7689 rear-view images of the hindlimbs from 2563 dogs. The basic characteristics of all dogs are described in Table 1. There were three pictures of the hind limbs from three different angles for each dog. To label the dataset, veterinarians examined the dog’s leg, touched the dogs’ medial patella, and diagnosed the presence and severity of MPL. We provided guidelines to each clinic for high-quality images and allowed them to obtain hindlimb images more accurately. The guidelines contain the dogs’ hip and leg locations, the distance from the camera, and brightness. In addition, information such as breed, age, weight, and presence of other diseases was also collected but not used in this study. In this study, no experiments were conducted on the animals, and hence, there is no legal issue of animal research ethics.

**Table 1:** Basic characteristics of all dogs.

	Dog, N	Image, n	Average age	Average weight (kg)	Most popular dog type (Top 3)	Previous PL* diagnosis	
						Yes	No
Total	2563	7689	5.52	5.59	1. Maltese (607)	117	2446
					2. Poodle (400)		
					3. Pomeranian (278)		
Normal	1072	3216	5.2	5.95	1. Maltese (221)	36	1036
					2. Poodle (206)		
					3. Pomeranian (147)		
Suspected^a^	732	2196	6.28	4.89	1. Maltese (154)	29	703
					2. Pomeranian (106)		
					2. Poodle (82)		
Stage 1 MPL	212	234	6.89	4.7	1. Maltese (72)	16	196
					2. Poodle (44)		
					3. Bichon (16)		
Stage 2 MPL	500	1500	5.32	4.67	1. Maltese (146)	31	469
					2. Poodle (67)		
					2. Pomeranian (59)		
Stage 3 MPL	44	132	6.85	4.35	1. Maltese (14)	4	40
					2. Pomeranian (9)		
					3. Chihuahua (7)		
Stage 4 MPL	2	6	9	6.5	1. Mix breed (1)	1	1
					2. Pekingese (1)		
Unknown^b^	1	3	1	6.7	1. Toy poodle (1)	0	1

^a^Suspected: Close to Stage 1 MPL but not yet.

^b^Unknown: inability to distinguish between normal and MPL. MPL, Medial Patellar Luxation.

PL*: Patellar Luxation.

**Figure 1:**
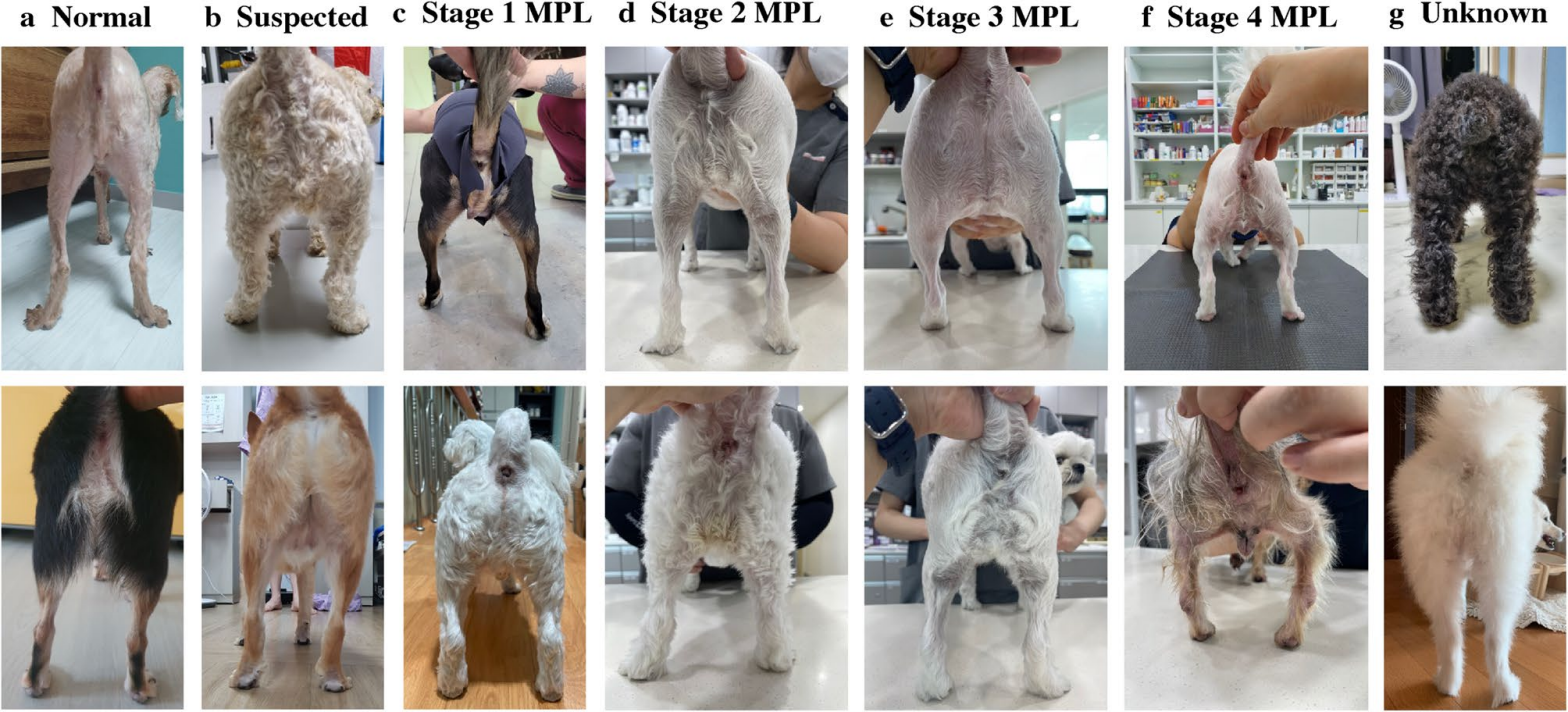
Representative cases of medial patellar luxation (MPL) for each Stage: Normal (**a**), MPL Suspected (**b**), Stage 1 MPL (**c**), Stage 2 MPL (**d**), Stage 3 MPL (**e**), Stage 4 MPL (**f**), Unknown (**g**). In MPL Suspected (B), compared to Normal (A) and Stage 1 MPL (C) Its bounded is unclear (2-Column).

The data collectors captured pictures from three different angles for each dog. We enrolled two professional veterinarians who did not participate in the experiment to select one of the three pictures of each dog that showed the best MPL independently. The veterinarian excluded cases in which the MPL was not visible because of low image quality or excessive noise. Figure 1 shows images of normal and stages 1, 2, 3, and 4 MPL. The two veterinarians checked the quality of the images independently so they could double-check each other.

### Data preprocessing

We filtered 2563 rear views of the hindlimb images from 7689 total images. Because CNN-based models take 224 × 224 RGB images as input^13^, we down sampled all images to 224 × 224 RGB images while considering their original shape ratio. By doing so, we aimed to prevent deformation and preserve the intended shapes of the original photos within the downsized images. To prevent overfitting, in which the deep learning model was focused on specific weights during the training process, we applied data augmentation to all training images. Data augmentation^14^ is a representative method for ensuring generality in the training process of a deep learning model.

Specifically, data augmentation included the following processes: (i) random horizontal/vertical flips, (ii) random affine with no rotation but randomly translation to left and right in the range of [0.1, 0.1] pixels (iii) color jitter with brightness: 0.5, contrast: 0.5, saturation: 0.5, hue: 0.5. Data augmentation was used only in the training process, and only images without any transformations were used in the testing model.

### Model architecture

**Figure 2:**
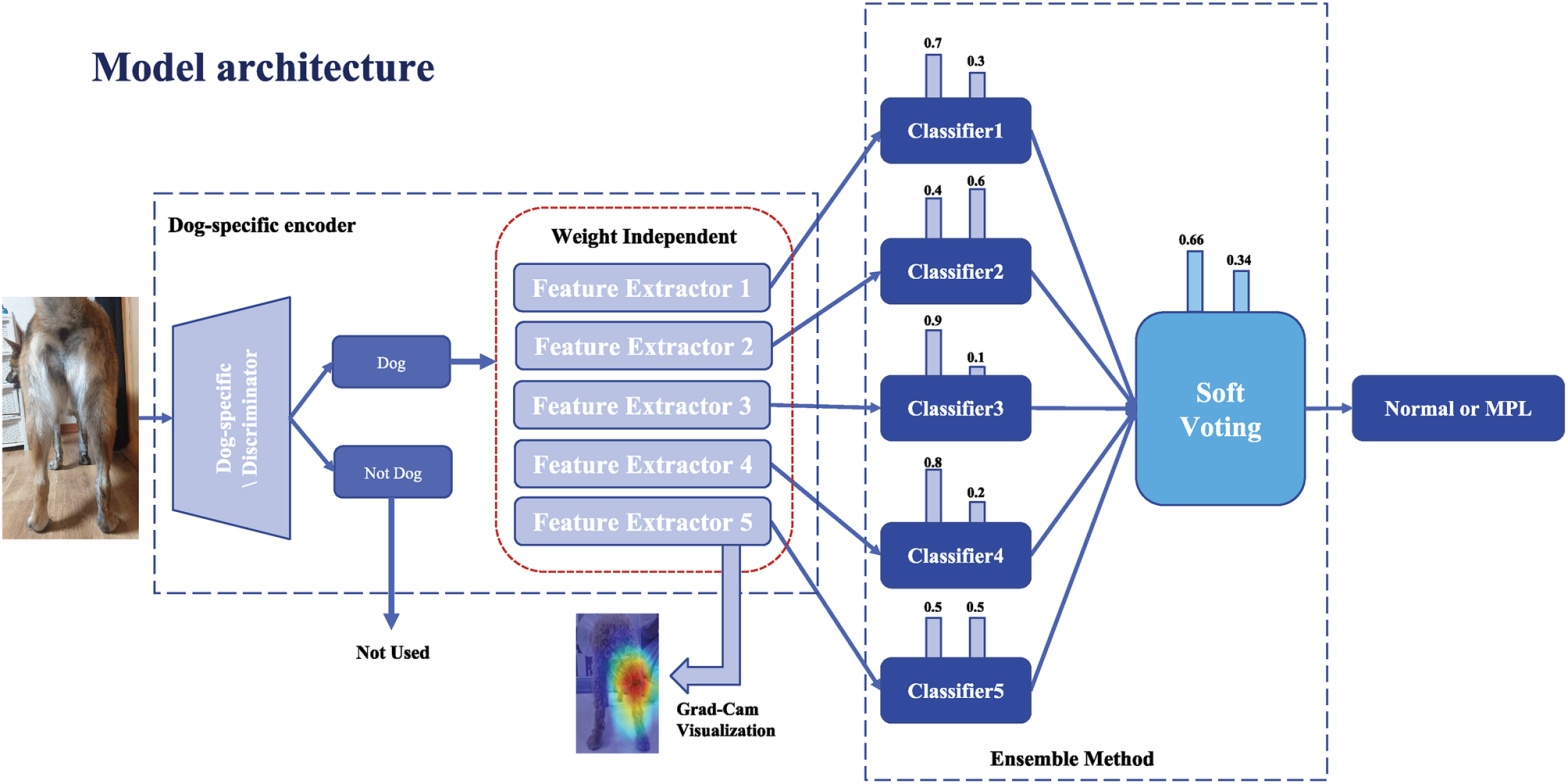
Architecture of the proposed Ensemble model based on ResNet18. Our proposed model consists of (i) a dog-specific discriminator, (ii) a dog-specific encoder (iii) an ensemble classifier. Specifically, a ResNet18-based dog-specific encoder consisted of an input layer, and 18 convolutional neural network layers with ReLU activation functions in five residual blocks with adaptive-pooling layers, an FC layer, and sigmoid. The final sigmoid activation function has been used to predict normal or Medial Patellar Luxation (MPL). (2-Column).

The proposed model comprised (i) a dog-specific discriminator, (ii) a dog-specific encoder, and (iii) an ensemble classifier. Figure 2 shows the architecture of the proposed model.

Since the input image may not be the rear-view image of the hindlimb or may be taken at an odd angle that can occur in single-image classification^15^, in the first stage, the dog-specific discriminator (Supplementary information 1) discriminates whether the input image is a dog’s hindlimb. The dog-specific discriminator consists of a one-class support vector machine (SVM)^16^ placed in the front part of the model to perform a role in discriminating whether it is a dog. The one-class SVM discriminator process is as follows. First, representation features were extracted from an input image, and the dimension of features was reduced with the principal component analysis** (**PCA)^17^. PCA is one of the most widely used dimensionality reduction methods that can transform high-dimensional data into low-dimensional data while preserving the distribution of the original data as much as possible. Consequently, we reduced 50,176 raw features to 200 principal components for each image using PCA. We then projected these 200 components to the vector space and found a decision line that could separate dogs and others. Moreover, by adjusting the threshold of the discriminators, our model selected high-quality dog images. Specifically, the discriminator filters out an image in which MPL cannot be distinguished owing to various noises and increases the accuracy and reliability of the model. This is enabled by the dog-specific discriminator, which identifies images as dogs. Images identified as dogs by this discriminator are then directed to the second stage, where they are processed by the feature extractor to extract image features.

In the second stage of the architecture, we built a dog-specific encoder with a Resnet18-based encoder that diagnoses MPL using a single rear-view image of the hindlimb. As shown in Table 2, we chose resnet18^18^ among other well-known CNN models as the baseline model because its accuracy was the best. Interestingly, in the CNN model, the performance of the model improved as the layer became deep; however, when the layer became too deep, the performance degenerated. This phenomenon is called the gradient-vanishing problem^19^. ResNet is a well-known deep neural network that can solve the vanishing gradient problem through residual learning using skip connections. This advantage of resnet18, with few parameters, demonstrated the best performance in the monotonous dog hindlimb image with residual learning. As shown in Table 2, the accuracy of ResNet18 was higher than ResNet 50, which had a deeper layer depth. As a result, our dog-specific encoder comprised 18 CNN layers in five residual blocks, followed by a rectified linear unit (ReLU). In addition, we used an adaptive pooling layer^20^ as the last pooling layer, which automatically adjusted the stride and kernel size to the input feature map. We applied the transfer learning method^21^ to reduce overfitting and to train the model faster. Specifically, we initialized 18 convolutional layers with pre-trained weights from the ImageNet dataset^22^. Because the rear-view image of the dog’s hind limb has a simpler structure than the other images and has a similar shape, fewer layers and learning parameters performed well. Consequently, our proposed model has 11,177,538 trainable parameters.

**Table 2:** Performance comparison between the proposed model and CNN model.

	Accuracy	Precision	Recall	F1-Score
Model	0.925	0.930	0.940	0.929
ResNet18	0.910	0.905	0.905	0.910
ResNet50	0.885	0.897	0.900	0.885
VGG16	0.870	0.830	0.810	0.870
VGG19	0.860	0.875	0.880	0.860
Inception_V3	0.905	0.900	0.900	0.905

Finally, we applied the ensemble method to the model’s training process to overcome image uncertainty. As shown in Fig. 2 we built five weight-independent classifiers, and each classifier made independent predictions with the same input image. The model made the final decision by soft voting on these five predictions. Soft voting^23^ selects the final predicted probability by averaging the prediction probabilities of the five classifiers for each class. Thus, the model can make fair decisions without bias or overfitting. It also supplements the need for more information on a single image.

### Grad-CAM visualization

We used Grad-Cam^12^ to visualize the region recognized as important in the image of the dog’s hind limb. The Grad-cam extracts representative features from the last layer output of the model and shows their importance using a heatmap. Interestingly, the regions represented by this heatmap are those that the model recognized as important in decision-making. We calculated the activated part using the gradient of the feature map that came out through the CNN layer. The resulting heatmap highlights the area of the image used in the classification of the model.

### Experiment setting

We performed fivefold cross-validation^24^ to train and evaluate the model. We randomly divided the dataset into five folds, trained the model four times, and tested the model on the remaining fold. This is because even if we shuffled data and randomly split it into five folds, certain class data can be gathered in one fold due to statistical probability. We trained our model using an ensemble learning method for each of the five folds. The average of the five test results obtained through the five classification validation processes was evaluated as the final performance of the model. We divided the data by dogs such that the same dog was not duplicated in the training and test data. To compare our model with other CNN models, we performed the same five-fold cross-validation for these CNN models with the same five data folds. All models, including our proposed model, were trained using a batch size of 32, epochs of 200, utilizing the Adam optimizer with a learning rate of 0.001.

To compare our model with that of five veterinarians, we selected 25 images of normal dogs and 25 images of dogs with MPL that were not used for training and testing the model. The data collector selected 50 images, and a professional veterinarian who did not participate in the experiment verified the image.

### Statistical analyses

Cohen’s kappa coefficient was used to rate the agreement level between the two veterinarians. We used a well-known Python library, Scikit-learn, to perform statistical analysis.

## Calculation

Performance of the proposed model was evaluated using the following metrics: accuracy, recall (also called sensitivity), F1 score, and precision (also called positive predictive value [PPV]). These metrics were calculated as follows:

1 $$\mathrm{Accuracy}= \frac{\mathrm{TP}+\mathrm{TN}}{\mathrm{TP}+\mathrm{FP}+\mathrm{FN}+\mathrm{TN}}$$

2 $$\mathrm{Precision}= \frac{\mathrm{TP}}{\mathrm{TP}+\mathrm{FP}}$$

3 $$\mathrm{Recall}= \frac{\mathrm{TP}}{\mathrm{TP}+\mathrm{FN}}$$

4 $$\mathrm{F1\,\,score}= \frac{2\times \mathrm{TP}}{2\times \mathrm{TP}+\mathrm{FP}+\mathrm{FN}}$$

where TP is the total number of images of dogs correctly diagnosed with MPL, FP is the total number of images of normal dogs incorrectly categorized as having MPL, FN is the total number of images of MPL that were improperly identified as normal, and TN is the total number of images of normal dogs that were misdiagnosed.

## Results

We contacted 30 private animal clinics and collected 7689 images of 2563 dogs from three different angles for each dog. The average age of the dogs was 5.52 years, and the average weight was 5.59 kg. Table 1 shows the characteristics of all dogs. We hired an experienced veterinarian to select and verify the best-quality image among the three pictures for each dog. Moreover, low-quality images were excluded. Consequently, 1072 normal dogs and 758 dogs with MPL were used to train and test the model.

### Model performance

Table 2 lists the accuracy of the proposed model and well-known CNN model. To compare the performance of the models, we utilized 1630 images from 972 normal dogs and 658 dogs with MPL for training and utilized 200 images form 100 normal dogs and 100 MPL dogs for testing the models. Our model, based on ResNet18, showed a 92.5% accuracy in detecting MPL. Its performance was better than that of VGG16 (87%), VGG19 (86%), ResNet50 (88.5%), and Inception-V3 (90.5%). Our model showed the highest accuracy among the well-known CNN-based models.

### Performance comparison with veterinarians

Table 3 shows the five veterinarians and the accuracy of the proposed model. The five professional veterinarians we recruited had an accuracy of 50–60% in diagnosing MPL by looking at only a single image. The average accuracy of these veterinarians was 55.2%, whereas that of our model was 90%. To conduct a fair experiment between our model and veterinarians, we selected 25 images of normal dogs and 25 images of dogs with the best quality for the comparison experiments, which were not used for model training. Therefore, the higher accuracy of the proposed model, even on 50 images that both veterinarians and the model had never seen, confirmed that our model had a higher accuracy in diagnosing MPL than experienced veterinarians.

**Table 3:** Performance comparison between the proposed model and human experts.

	Accuracy	Sensitivity	Specificity	Precision	F1-score
Model	0.90	1.00	0.80	0.83	0.91
V1	0.50	0.28	0.72	0.50	0.36
V2	0.52	0.44	0.60	0.52	0.48
V3	0.58	0.44	0.72	0.51	0.61
V4	0.56	0.36	0.76	0.60	0.45
V5	0.60	0.44	0.76	0.65	0.52
V average^a^	0.552	0.392	0.712	0.556	0.484

^a^V average denotes the average of five veterinarians. V, veterinarian.

**Figure 3:**
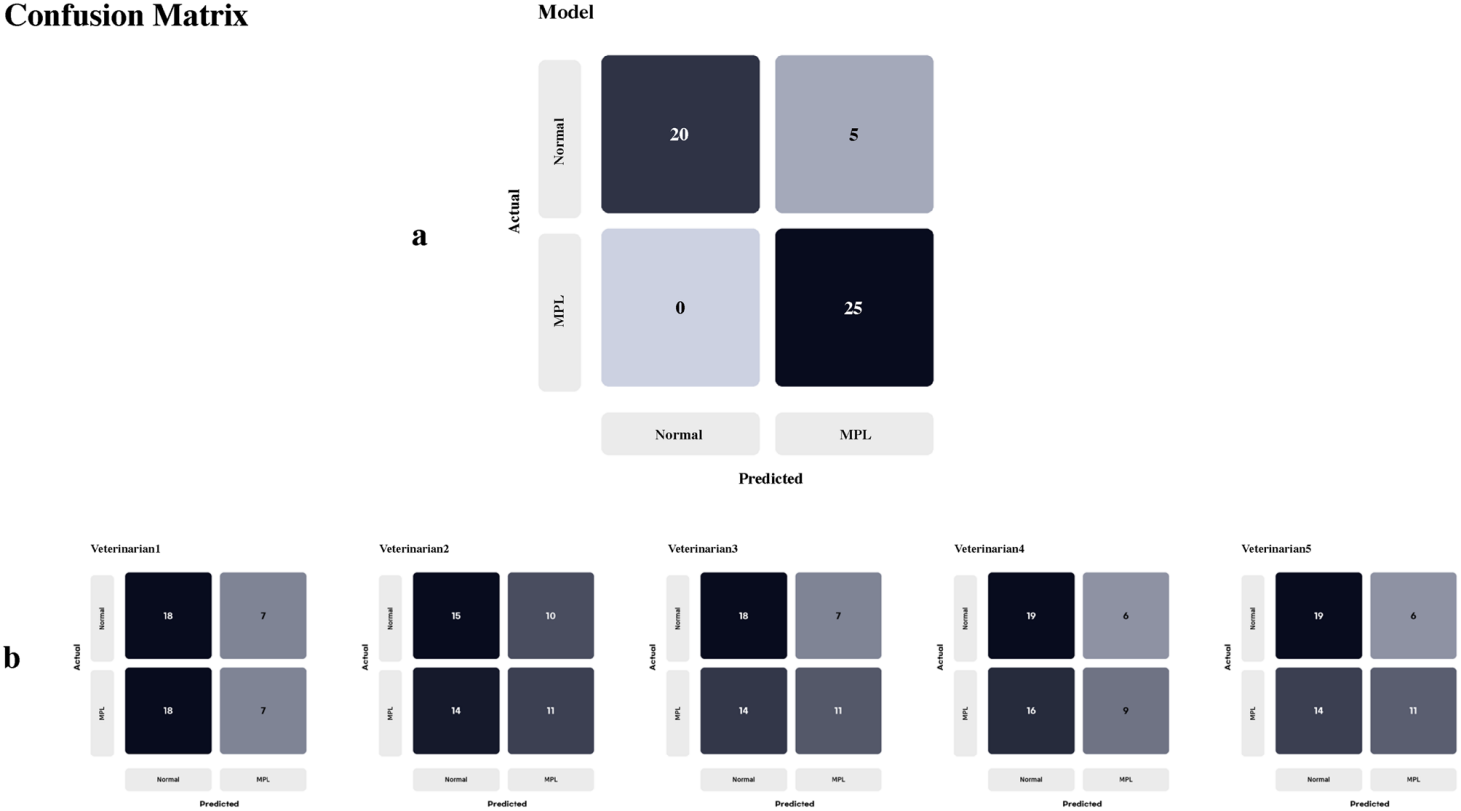
Comparison confusion matrix between the proposed model and the veterinarians. (**a**) Proposed model’s confusion matrix. (**b**) Five veterinarians’ confusion matrix. V1, 2, 3, 4, 5 denotes 5–10 years experienced Veterinarians. (2-Column).

We analyzed the cases in which our model was correct and those in which it was not. Figure 3 shows the confusion matrix of our model and that of the five veterinarians. The average specificity and sensitivity for veterinarians were 71.2% and 39.2%, respectively. As shown in Fig. 3b, the high specificity and low sensitivity indicate that veterinarians diagnosed most cases as normal dogs. Conversely, as shown in Fig. 1a, our model never misclassified MPL as normal but only misdiagnosed normal dogs as having MPL.

**Figure 4:**
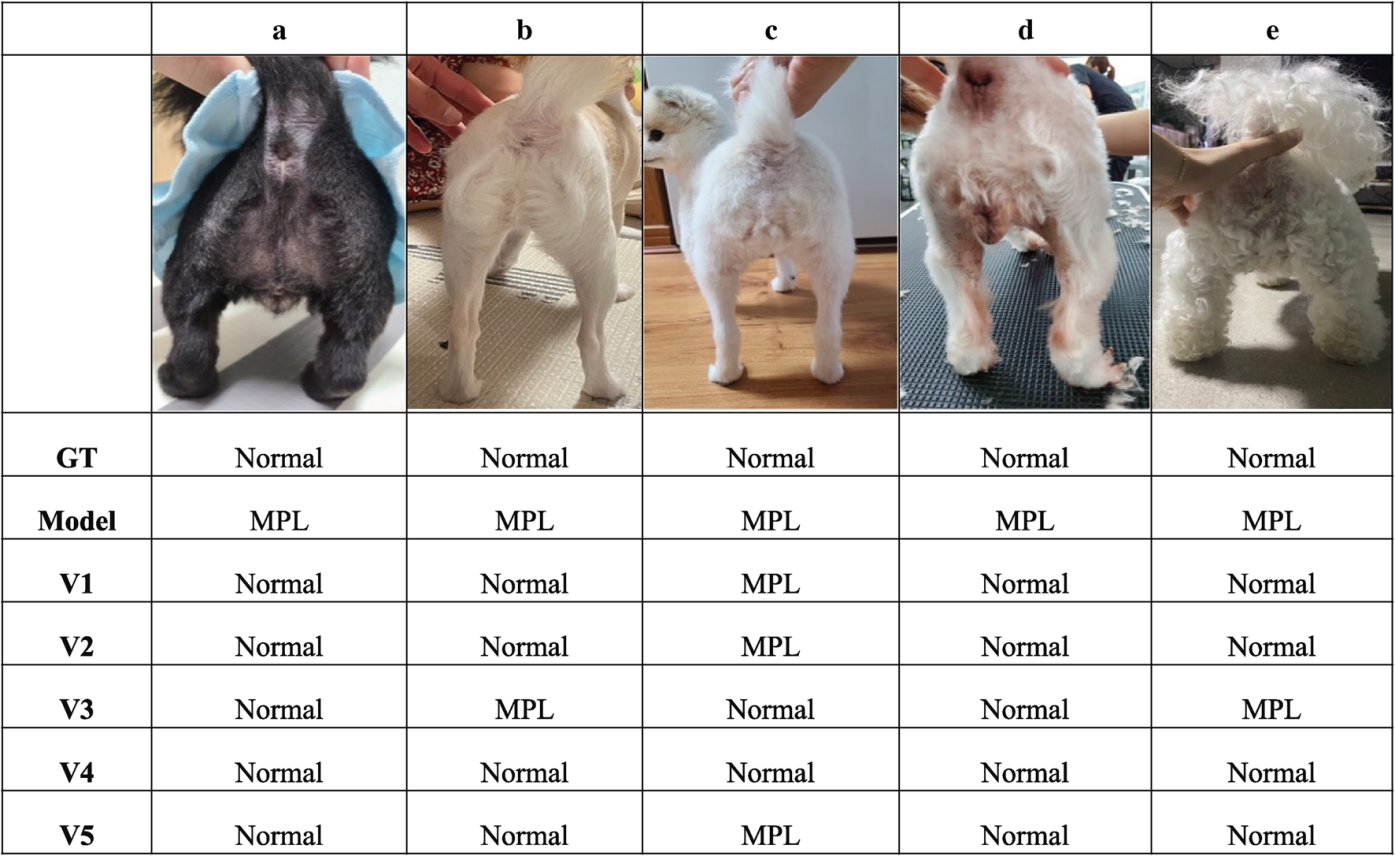
Comparison of medial patellar luxation (MPL) detection between the proposed model and human experts. The figure displays five instances where the proposed model exhibited incorrect detection results. The ground-truth (GT) annotations and the five veterinarians’ (V1–V5) expertise were used for benchmarking and comparison with human detection performance. (2-Column).

Figure 4 shows five cases where our model made an incorrect prediction. Although the real answer was normal in all five cases, the model predicted all of them as MPL. As shown in Fig. 4e, the curvature of the legs is not observed clearly because of the dog’s fur. Additionally, as shown in Fig. 4a, b, and d, the dog’s legs seemed bent even though the case is normal. These noises caused our model to predict incorrectly.

The Kappa coefficients between the first and fifth veterinarians and the third and fourth veterinarians were 0.39, 0.50, demonstrating moderate agreement (*P* < 0.001) in diagnosing MPL. In contrast, the Kappa coefficients between the third veterinarian and the model and the fifth veterinarian and the model were 0.17 and 0.14, respectively, demonstrating slight agreement (*P* < 0.001) in diagnosing MPL.

### Visualization of model’s decision-making process with Grad-Cam

**Figure 5:**
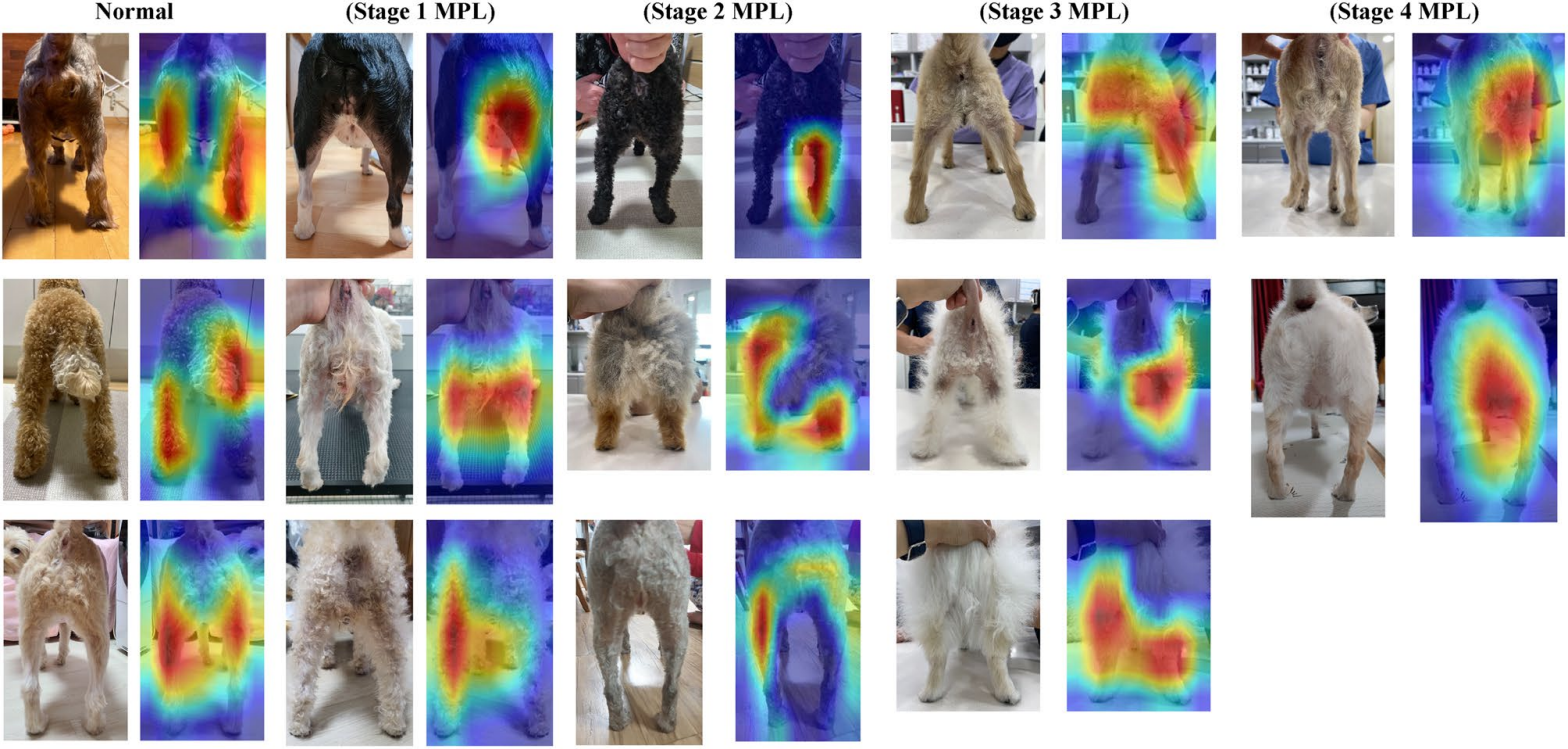
Heat maps for the proposed model’s predictions by gradient weighted class activation mapping (Grad-CAM). Grad-cam calculates the representative region in the image while the training process. Grad-cam shows important parts of the model’s decision-making process with a heat map. (2-Column).

Figure 5 shows a feature-based heat map provided by the gradient-weighted class activation mapping (Grad-CAM)^11^. The area highlighted in the Grad-Cam image is the medial patella. When MPL occurs, the corresponding part is bent^12^. This heat map confirms that our proposed model diagnoses MPL while observing the actual medial patellar location.

## Discussion

We aimed to build a deep-learning model that accurately diagnoses MPL using a single rear-view image of the hind limb. Many previous studies have focused on improving the performance of deep neural networks by drawing object outlines or using segmentation algorithms^25^. A representative study improved object detection accuracy by proposing an accurate edge detector using richer convolutional features (RCF) of images^26^. Therefore, by using a segmentation algorithm, the performance of the proposed model will also be more accurate. However, our study is the first attempt to accurately discriminate the presence or absence of MPL, even with a single picture taken with a smartphone. Our model also showed higher accuracy than other well-known CNN models and professional veterinarians without using a segmentation algorithm. Following the experiment, we conducted a survey of veterinarians and found that less-experienced professional veterinarians who have never diagnosed MPL by examining a single image have lower accuracy compared to the proposed model.

There are two major image problems when developing a model for diagnosing MPL with a single rear view of the image. First, the input image could not be an image of a dog or of low quality; second, a single image could not contain enough information to detect the MPL. These problems are referred to as image uncertainty^27^. To build a stable model against image uncertainty, we added a special dog-specific discriminator to the front of our deep-learning system and trained our model using the ensemble method. The dog-specific discriminator distinguishes dogs from backgrounds by tuning the threshold; thus, the model can focus on dogs specifically. To take a picture of a dog’s hind limb, the person must hold the dog. Thus, the person or background is inevitably included in the image as a noise. Therefore, the discriminator automatically discriminates dogs from others; hence, only trainable images are fed into the model’s input. Moreover, we used the ensemble method^28^ to overcome the lack of information in a single image. The ensemble method yields more accurate and stable predictions by generating multiple classifiers and combining these predictions. A single model’s prediction can easily overfit; however, the ensemble method can prevent overfitting by combining the independent predictions of several classifiers^29^. We trained the proposed model using the ensemble method and developed a robust model that can overcome the uncertainty of a single image.

Our study had several limitations. First, when diagnosing MPL, only a single image taken under strict standards was considered as the input of the model. We provided guidelines to the data collectors and let them take rear-view pictures of dog hindlimbs under the same conditions. The guidelines are as follows: when taking photos of the dog’s hindlimb, one person should hold the dog, while another should take a horizontal picture of the dog’s buttock, with the dog’s buttocks, paws, kneecaps, and medial patella being visible. However, in the real world, people take photos of the dog’s hindlimb from various angles, and the severity of MPL may differ depending on the angles and sights. Hence, diagnosing MPL using only a single image taken in the real world is limited. However, because our study used only images taken under specified conditions, the MPL was well revealed using only a single image. Second, we used only a single image formed in front of the hindlimb without considering all images taken from various angles. Thus, in our study, information on the MPL from multiple angles was not considered. When veterinarians diagnose MPL, they also examine the extent to which the patella is bent from various angles. The model would be more accurate if trained using multiple images taken from different angles. Thus, in future studies, we can develop a multi-image input model based on 3D- CNN^30^, multi-instance model^31^, or attention model^32^ to increase the accuracy of MPL diagnosis by considering all images taken from various angles and creating a robust model. Third, noise exists in the image. Dogs have very different hair shapes depending on the breed. Because of this hair, there were complex cases for the model to determine the presence or absence of MPL. Figure 4 shows the incorrect decisions of the model for classifying hairy dogs. Based on a study of estimating the shape of the face by finding the key points of the face with a deep neural network^33^, this problem can be solved by drawing the dog’s outline or capturing the key points of the joints and calculating the bent patella angles. Lastly, MPL occurs more often in smaller and older dogs^34^, and the model diagnoses MPL using only one rear-view image of the hindlimbs without considering other dog information. Figure 3 confirms that veterinarians cannot diagnose PL using just one hind leg image for diagnosing MPL. Moreover, we interviewed veterinarians after the experiments, and all the veterinarians answered that it was challenging to diagnose MPL by looking at only a single image because veterinarians had never diagnosed MPL by looking at only one image. Thus, as a follow-up study, we are trying to create a model for diagnosing patella dislocation considering the hind leg image and the dog’s age, weight, sex, and breed. We expect the proposed model to be more robust by providing additional information as model input.

In summary, we created a deep learning system that diagnoses MPL with 90% accuracy using only a single image of the hind limb. We attached a dog-specific discriminator to the front of our model to discriminate the dog regardless of any image so that the model could distinguish whether the input image was a dog or not. In addition, to verify the robustness, we applied an ensemble learning method to solve various problems in diagnosing patellar dislocation using only a single image. The proposed model can support veterinarians in diagnosing patellar luxation.

### Supplementary Information

Supplementary Figures.

## Data Availability

The data are not available for public access due to ethical restrictions and veterinary hospital privacy concerns, but are available from the corresponding author on reasonable request.
