# Supplementary figures and images for: Detecting medial patellar luxation with ensemble deep convolutional neural network based on a single rear view image of the hindlimb
Source: Sci Rep. 2023 Oct 10;13:17113. doi: 10.1038/s41598-023-43872-7 (PMC10564780; doi:10.1038/s41598-023-43872-7)

# Model architecture

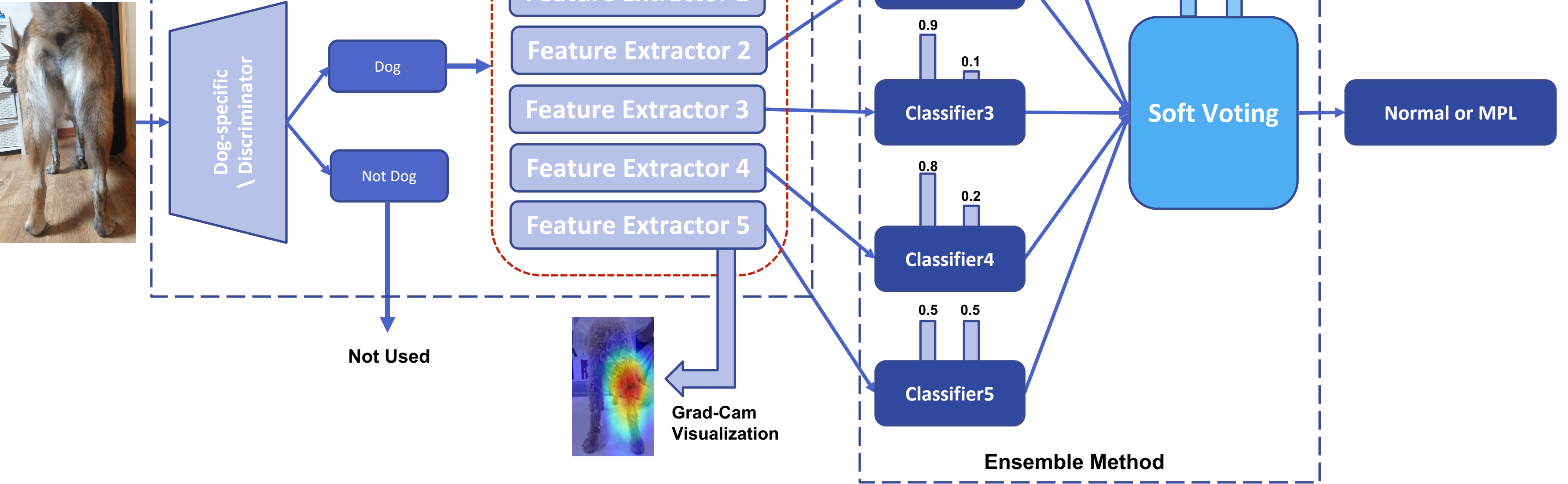

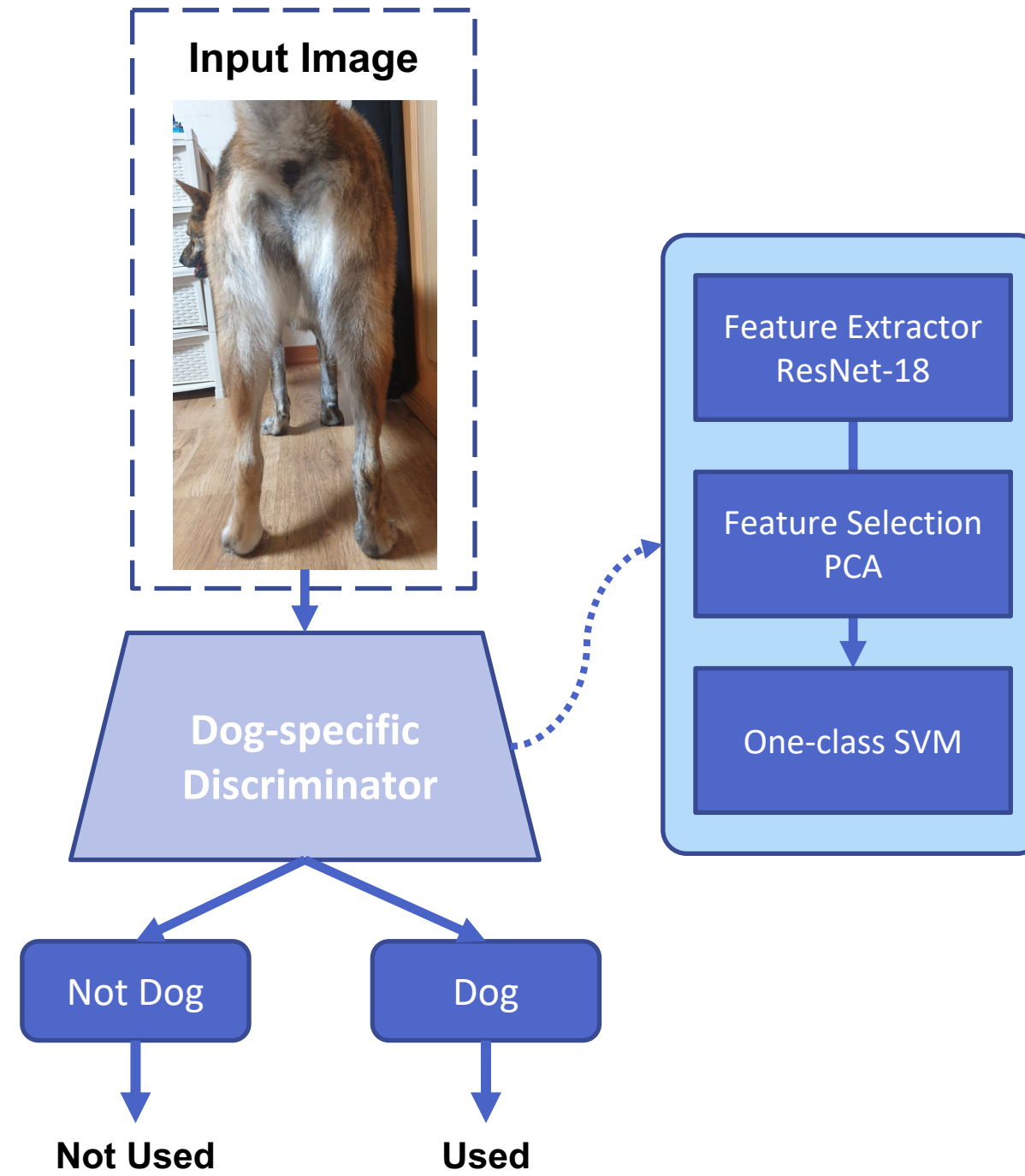

# Model architecture

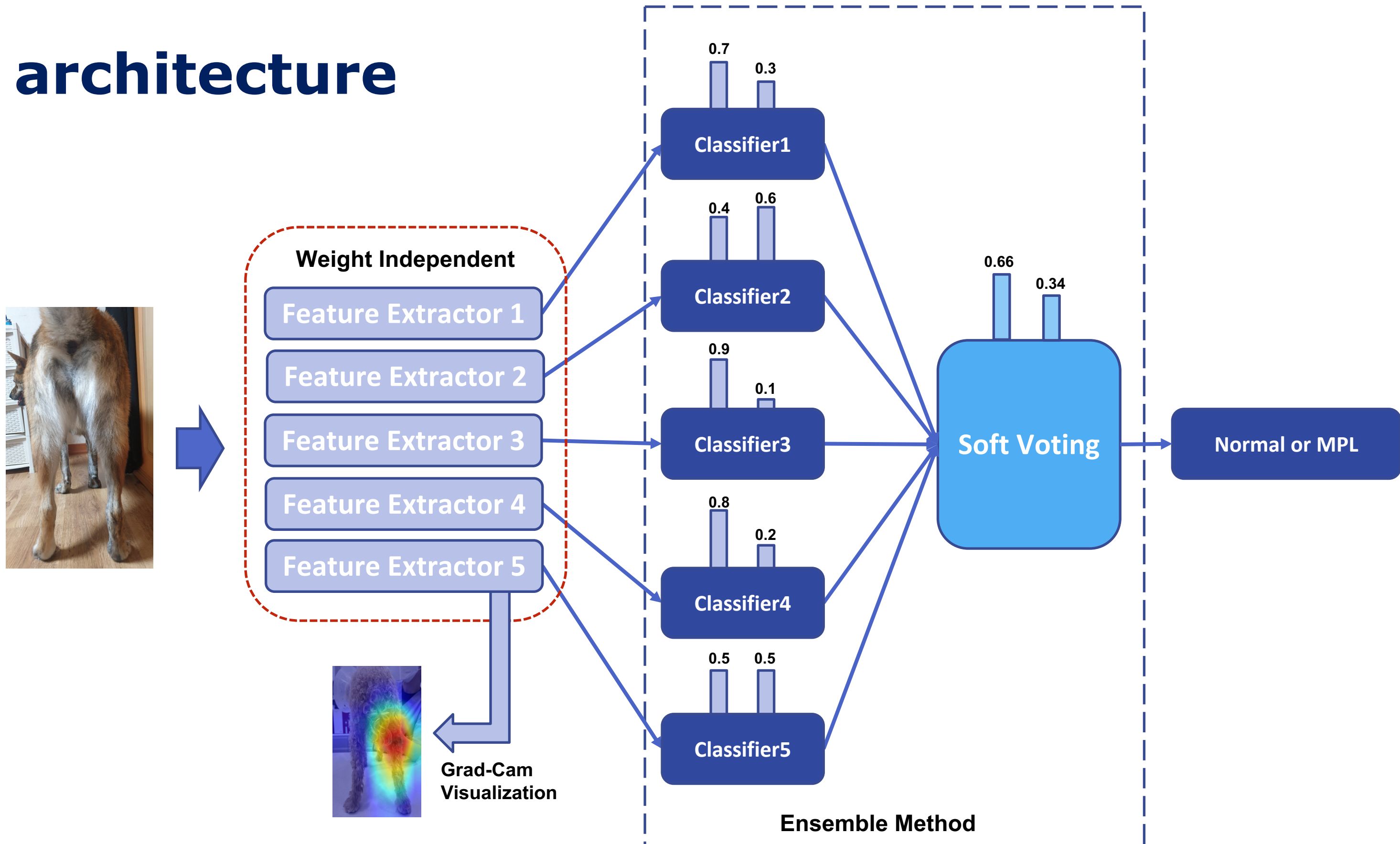

Supplement: Supplementary file 1 — Supplementary Figures. [file 41598_2023_43872_MOESM1_ESM.pdf]
